# A Multi-Stage Method for Connecting Participatory Sensing and Noise Simulations

**DOI:** 10.3390/s150202265

**Published:** 2015-01-22

**Authors:** Mingyuan Hu, Weitao Che, Qiuju Zhang, Qingli Luo, Hui Lin

**Affiliations:** 1 Shenzhen Research Institute, The Chinese University of Hong Kong, 2nd Yuexing Road, Nanshan District, Shenzhen 518057, China; E-Mail: huilin@cuhk.edu.hk; 2 Institute of Space and Earth Information Science, The Chinese University of Hong Kong, Fok Ying Tung Remote Sensing Building, CUHK, ShaTin, N.T., Hong Kong; 3 Department of Geo-information Processing, Faculty of ITC, University of Twente, Hengelosestraat 99, Enschede 7500 AE, The Netherlands; E-Mail: zhangqiuju0928@gmail.com; 4 Center of Remote Sensing, Tianjin University, No. 92, Weijin Road, Tianjin 300072, China; E-Mail: luoqingli2003@163.com

**Keywords:** participatory sensing, noise simulation, virtual partition, spatio-temporal data organization

## Abstract

Most simulation-based noise maps are important for official noise assessment but lack local noise characteristics. The main reasons for this lack of information are that official noise simulations only provide information about expected noise levels, which is limited by the use of large-scale monitoring of noise sources, and are updated infrequently. With the emergence of smart cities and ubiquitous sensing, the possible improvements enabled by sensing technologies provide the possibility to resolve this problem. This study proposed an integrated methodology to propel participatory sensing from its current random and distributed sampling origins to professional noise simulation. The aims of this study were to effectively organize the participatory noise data, to dynamically refine the granularity of the noise features on road segments (e.g., different portions of a road segment), and then to provide a reasonable spatio-temporal data foundation to support noise simulations, which can be of help to researchers in understanding how participatory sensing can play a role in smart cities. This study first discusses the potential limitations of the current participatory sensing and simulation-based official noise maps. Next, we explain how participatory noise data can contribute to a simulation-based noise map by providing (1) spatial matching of the participatory noise data to the virtual partitions at a more microscopic level of road networks; (2) multi-temporal scale noise estimations at the spatial level of virtual partitions; and (3) dynamic aggregation of virtual partitions by comparing the noise values at the relevant temporal scale to form a dynamic segmentation of each road segment to support multiple spatio-temporal noise simulations. In this case study, we demonstrate how this method could play a significant role in a simulation-based noise map. Together, these results demonstrate the potential benefits of participatory noise data as dynamic input sources for noise simulations on multiple spatio-temporal scales.

## 1. Introduction

In urban areas, noise pollution has become a serious environmental problem that adversely impacts the health of the population and degrades its quality of life [1–3]. To understand the effects of noise pollution, some standard data collection practices have been performed to support noise simulations, which serve as input for future decision-making [4,5]. Currently, the major sources of noise data used in the simulations are calculated from daily statistics regarding traffic volume or collected at predefined locations. These discrete collections are usually expensive and time consuming, which results in low update frequency for noise simulations (e.g., 5 years for the UK [6]) and makes it difficult to analyze the noise impact at different spatio-temporal granularities.

Advances in Information and Communication Technologies (ICTs) especially sensors and sensor networks are producing a quite different urban environment that we have experienced hitherto [7], leading to a transformation of the cyber and real cities into smart cities [8]. As sensors can be integrated into nearly all parts of the real world, sensor-based sensing is at the heart of smart cities [9]. Hereinto, sensor-rich smart phones have made possible the recent birth of the mobile sensing research area [10]. A mobile sensing system (MSS) could be simply regarded as a user-level application (app) running on the phone to read data from an internal sensor in the phone, or external sensors in the wireless sensor network (WSN) and then reporting the sensed data to the Web. As a type of environmental-centric and human-based MSS, mobile device-based participatory sensing [11] has been gradually applied for collection of actual noise data with multiple granularities in space and time to improve the update frequency and reduce the cost of data collection for noise mapping. However, participatory data are generally contributed by volunteer participants at arbitrary locations and times in most cases [12]. Consequently, collected samples of noise measurements are randomly distributed in space and time, thus making processing and management of these data difficult, especially for the purpose of supporting noise simulations. Therefore, effective organization and management of these participatory data are needed to further support the data input for noise simulations to improve noise mapping [6,13].

Within the context of the sensors and smart cities, the purpose of this study was to provide a methodology to bridge noise simulations and participatory noise monitoring and thus to facilitate more rational simulation results. We first present a multi-stage method to coordinate participatory noise data. In this method, the granularity of the noise data on road segments can be dynamically refined to provide a reasonable spatio-temporal data foundation to support noise simulations. The multi-stage method includes three stages of spatial matching, dynamic estimation of noise data at multi-temporal scales and dynamic segmentation of road segments based on spatio-temporal aggregation of participatory noise measurements. Taking the campus of the Chinese University of Hong Kong as a case study, dynamically processing, organizing and refining noise information on road segments were performed on different temporal scales. Furthermore, a dynamic spatio-temporal database was formed with the support of participatory sensing approach; this database can be fed into simulations as the ground truth to improve noise mapping. A comparison of the results demonstrated the potential benefits of participatory noise data as dynamic input sources for noise simulations at multiple spatio-temporal scales, especially for reflecting the short-term local variations in noise pollution.

To this end, this paper was organized in the following manner: the related works are introduced in Section 2. Section 3 provides the methodology for coordinating the participatory noise data to form a dynamic spatio-temporal database to support noise simulations. Experimental results and discussions are presented in Section 4. Finally, Section 5 provides the conclusions of this study and commons on future prospects.

## 2. Related Works

Noise simulations can help to obtain global trends of the urban soundscape and to provide an indication of the actual citizen exposure to noise levels [14]. Many cities in developed countries have applied noise simulations in noise assessments [4,15,16]. Currently, two main ways of collecting the input noise data exist: using sound level meters to perform field measurements at predefined locations and estimating daily traffic volumes. For field measurements, the conventional process of collecting noise data is expensive and time-consuming. For example, Madrid's Environmental Administration operates the largest urban noise-monitoring network, which is based on 4395 measuring points [17]. Such simulations can only provide the general noise conditions of an entire region during a time period; it is difficult to reflect short-term, local variations in noise pollution, particularly for accidental pollution peaks. The second method, which uses estimates of daily traffic volumes, generally relies on outdated statistics for traffic-related noise from road, rail and air traffic, and it is difficult to reflect the up-to-date noise levels in real scenarios. For example, a map from January 2013 may rely on outdated traffic volume statistics from 2008. Thus, because of the limited number of data measurements and missing noise sources, inaccurate simulation results are inevitable when using existing methods, despite the use of accurate landscape models and simulations. Moreover, increasing the amount of data collected by using more sound level meters and manpower is too expensive and does not scale [18].

The concept of participatory sensing was first introduced by Burke *et al.* [11] and has a wide range of applications, such as monitoring the performance and environment of a cyclist [19] and predicting the arrival time of a bus [20]. The mobile sensors carried by people (e.g., microphones and GPSs [21]) can be organized as external sensors in a WSN; these sensors have the potential to monitor and estimate noise pollution [22]. However, the uncontrollable procedure of data collection leads to uncertainties in the monitoring tasks and had an adverse effect on the management/use of participatory sensing data [12,13]. To guarantee a high level of trustworthiness for sensed data, two types of efforts are important here. On the one hand, following the framework of human-centric wireless sensor networks (HWSN) [23], participatory sensing-based data collection is allowed to present the right tasks to the right participants; data gathering is triggered on-demand by a user request and matched with the suitable monitoring tasks. In this framework, human-based mobile sensors can act as the nodes of the HWSN, thereby reducing the energy consumption of the nodes and protecting the privacy of users' activities and locations. On the other hand, participatory sensing can be performed as measurement campaigns that let volunteers measure data along a fixed route at predefined times to minimize data redundancy and avoid uncertain data; however, this method limits the possibility for widespread use of participatory sensing data. NoiseTube [24] and Ear-phone [6] are two notable participatory sensing mobile applications that have been used to generate collective noise maps via aggregation of volunteer measurements. Such participatory sensing applications specialize in collection and visualization of noise data, and the noise data are directly organized according to sequences of spatio-temporal points to facilitate representation of the raw data on maps [25,26]. However, considering that the participatory noise measurements are collected randomly in space and time, and that the data sets can be sparse or incomplete, more samples and more sophisticated statistical methods are required to produce credible results, especially for the purpose of supporting noise simulations. Some supplementary technologies to enhance the potential of the available data have been proposed, Examples include the spatio-temporal index of mobile data [27], generalizations from trajectory data for efficient pattern discovery [28], and data aggregation to protect privacy [29]. The ear-phone project has particularly investigated the feasibility of two sensing strategies, namely the projection method and the raw-data method, for reconstructing the complete noise data from incomplete samples [6]. Although such formal methods can take advantage of the availability of crowdsourcing sensors to estimate the missing data [30], they did not explore the spatio-temporal correlations among the participatory data that may be lost due to the inappropriate approximations of the spatial and temporal properties of the data. For example, noise measurements from participants were distributed unevenly with variable densities, and their values may differ from each other at the same location but at different time periods. In this regard, more focus should be placed on organizing noise information with multiple spatiotemporal granularities to support simulated analysis of the uneven spatiotemporal coverage of noise pollution. Moreover, previous methods have rarely taken participatory noise data as dynamic input sources for noise simulations on multiple spatio-temporal scales. An effective data coordination method would provide statistics obtained from measurements and extract local features with high granularity in space and time, which would further ensure well-organized results to support official noise simulations.

As the trend of “combining networked sensors with dynamic information flows into our daily lives” is becoming more feasible and affordable [31], a merging of professional simulation and participatory monitoring methods can be valuable under specific circumstances, and can benefit both citizens who are progressively raising people's awareness of the environmental issues at hand and official assessments [32]. Given the above-mentioned facts, the purpose of this study was to provide a methodology to bridge noise simulations and participatory noise monitoring and thus to facilitate more rational simulation results. The main idea was to effectively organize the participatory noise data in a manner such that the granularity of the noise features on road segments can be dynamically refined, and then provide a reasonable spatio-temporal data foundation to support noise simulations.

## 3. Methodology

**Figure 1.**
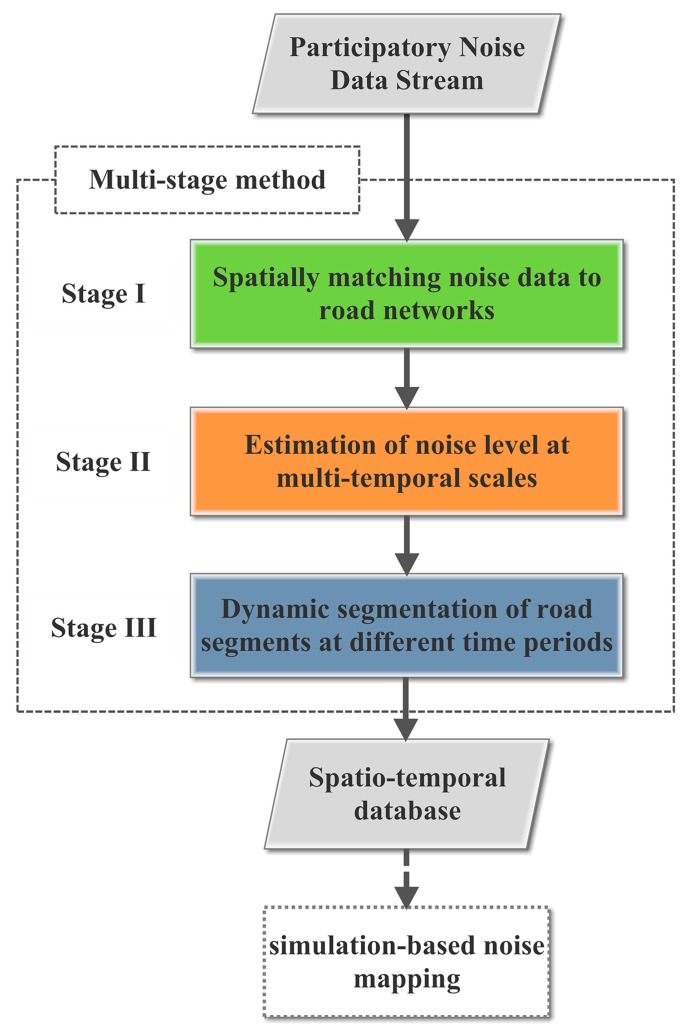
Framework of the multi-stage method.

In this section, we first introduce a multi-stage method for coordinating the participatory noise data. In this method, there are three stages: spatial matching; dynamic estimation of noise data at multi-temporal scales and dynamic segmentation of road segments based on the spatio-temporal aggregation of participatory noise measurements, in terms of dynamically processing, organizing and refining noise information on each road segment at different temporal scales; and construction of a dynamic spatio-temporal database with the support of participatory data-based noise simulations (see Figure 1).

### Stage I: Spatially Matching Noise Data to Road Networks

**Figure 2.**
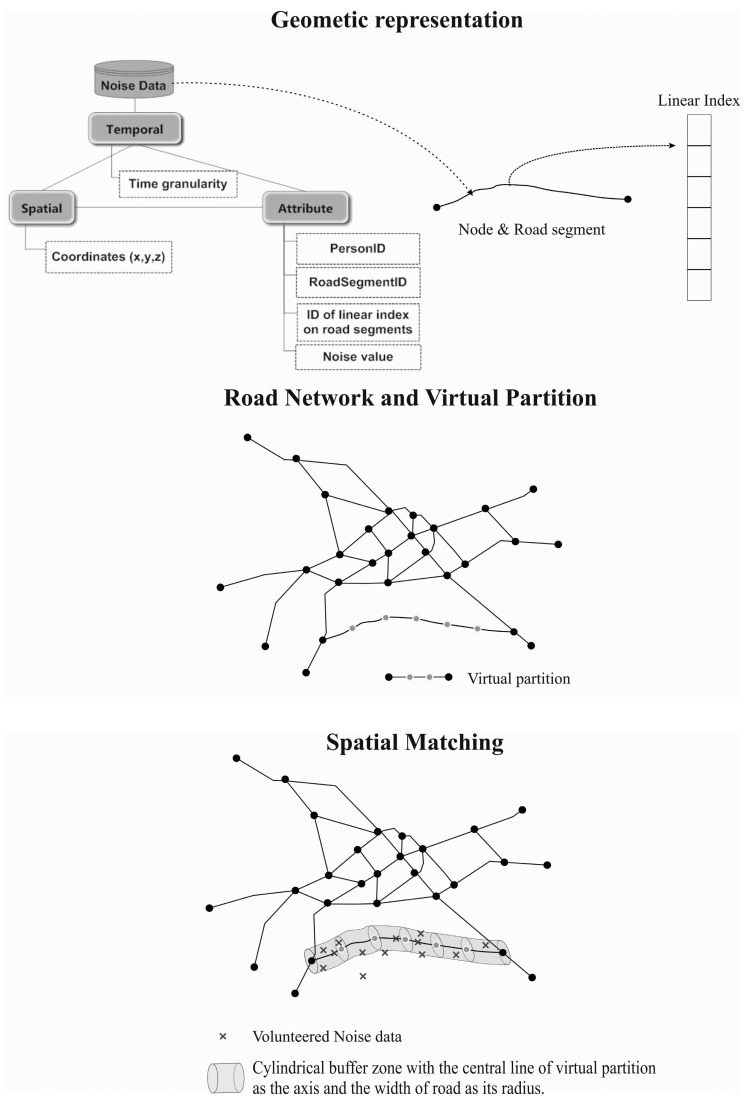
Spatial positioning components: geometric representation, semantic road networks, and spatial matching.

The basic spatial positioning component has three hierarchies, namely, geometric representation, semantic road networks, and spatial matching (see Figure 2).

The geometric representation hierarchy refers to the basic structures of geometric primitives to support the construction of road networks. First, the noise data collected dynamically were abstracted as a multi-dimensional feature, which included spatial (3D coordinates), temporal, and attribute dimensions. The corresponding data structures included PersonID, RoadSegmentID, ID of the linear index on road segments, X, Y, Z, dB(A) value, and Time. To spatially match the noise data to a road network, the structure of the arc-node can be used as the basic geometrical expression of road networks. Furthermore, the linear index is organized to further subdivide a road segment (arc) into more detailed divisions (virtual partitions) to link it with efficient noise data collection.

The semantic road network hierarchy includes the road network, road segments, and virtual partitions and refers to a topological description of road networks. The traditional model structure of a road network at the semantic level is road network-road segments. We refer to further partitions of road segments as virtual partitions (Figure 2). Specifically, a virtual partition in geometry refers to an initial partition of a road segment at equal geometric intervals, and the partition associates noise values at different temporal scales. For example, suppose that noise data were collected every second in which an ordinary person can walk a distance of 0.75–0.8 m; then, an initial virtual partition with a spatial granularity of 0.75–0.8 m can be generated to match the noise data. Thus, the road segment was subdivided into multiple virtual partitions to refine the noise data on a microscopic level. This partition further provided the basis for the dynamic estimation of noise values on the same road segment, which helped to identify spatial-temporal differences in noise values on the segment.

The spatial matching hierarchy helps to integrate dynamic noise information in a linear measure (e.g., virtual partition) into base road networks and refers to the process of spatial matching of the locations at which noise data were collected with their adjacent road segments (virtual partition). Considering the noise data near the road as the data source, the spatial relationships between dynamically collected noise data and the nearest roads must be established. The first step was to build a cylindrical buffer zone with the central line of the road (virtual partition) as its axis and the width of the road as its radius. The next step was to perform buffer analysis (which is the most commonly used method in the GIS field) to establish the spatial inclusion relation between the locations at which the noise data were collected and the cylindrical buffer zone. Thus, it enabled multiple sets of noise information to be associated with any portion of a linear road segment. In addition, invalid data (e.g., if the distance is too far from the buffer zone of the road central line) could be removed to reduce the memory burden.

### Stage II: Estimation of the Noise Level at Multi-Temporal Scales

**Figure 3.**
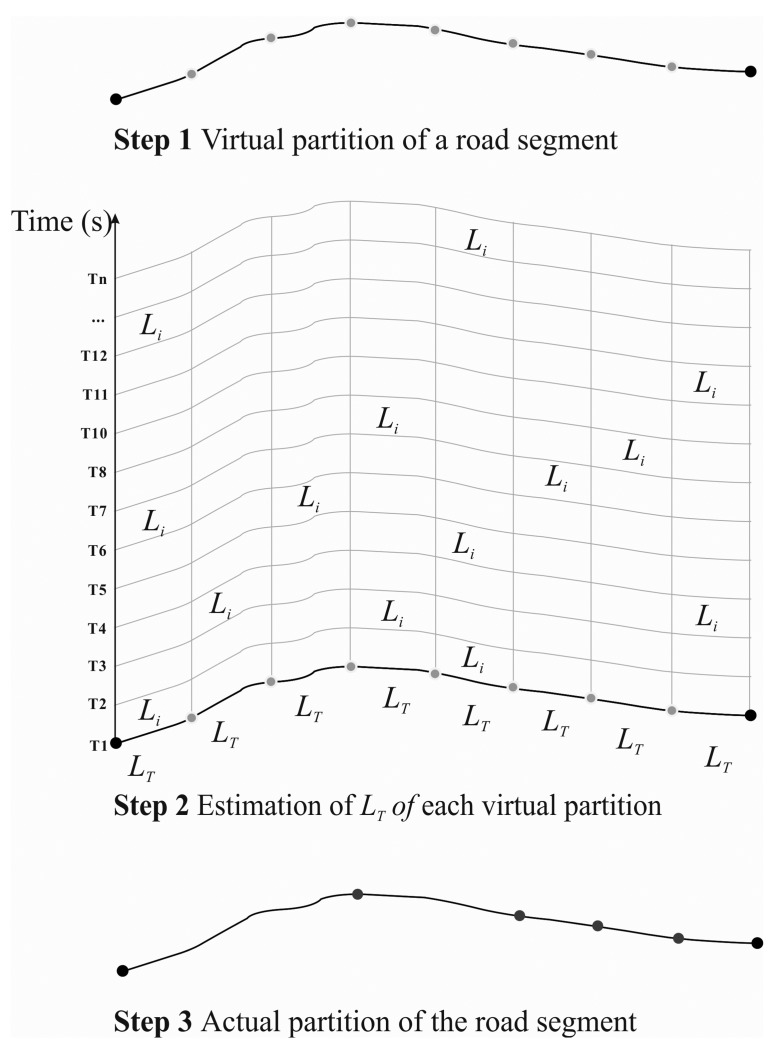
The process of dynamic estimation of noise levels at multi-temporal scales.

Noise information found at the same location can be a noise value at one time stamp or a set of values during a time period, which could be used to estimate the noise values at different temporal scales, such as seconds, minutes, hours, workdays, months, and years. To estimate each virtual partition of a road segment (Figure 3), the noise level at a given time period can be calculated by combining noise contributions from the participatory measurements and the initial traffic volume of the road segment using the following equation:

(1) $$L_{\text{T}}=10\times \log\left(\frac{1}{N_{T}}\times \left(\sum_{i=1}^{n}10^{\frac{L_{i}}{10}}+\left(N_{T}-n\right)\times 10^{\frac{L_{de}}{10}}\right)\right),\text{dB}(\text{A})$$

The equation used here is derived from the ISO 9612:2009. Noise levels on different timescales for all virtual partitions were estimated accordingly. Here, *L_T_* is the predicted noise level for a specific period of time (T), and T refers to the different scales (e.g., hours, days and months). *N_T_* refers to the ideal total counts of noise acquisition during a given time period; it can be calculated as the time period divided by the time intervals of the dynamic acquisition of noise measurement. *n* refers to the actual total counts of the data measurement during the given time period. However, it only takes valid measurements into account. *L_i_* refers to the value of the noise measurement at the time i, where i = 1,2,3,…, *N_T_. L_de_* refers to the default value for the noise data. It was used to fill in the gaps of data collection, *i.e.*, when no data were collected at a specific time. *L_de_* could be obtained from the initial noise database (see Section 4 for more details), which was calculated using the traffic volume of the road segment, or derived from actual measurements obtained in fieldwork.

### Stage III: Dynamic Segmentation of Road Segments in Different Time Periods

The final stage of the calculation process was to arrive at the actual partition of the road segment at different time periods, which formed the dynamic segmentation of each road segment based on merging of adjacent virtual partitions via a comparison of the noise values at the relevant temporal scale. According to the description of a perceptible change in noise under normal conditions [33], we selected the minimum perceptible difference (3 dB(A)) as the justified threshold value to partition the estimated noise values. The actual partition process is described in the algorithm presented in Table 1:

As shown in Figure 3 and Algorithm 1, a set of virtual partitions in one road segment is indicated by P = [P_1_, P_2_, P_3_, …, P_n_]. A noise set, which is expressed as V = [V_1_, V_2_, V_3_, …, V_n_], was then built using the selection of noise values in a required time period. The temporary aggregation A_temp_ was used to establish an actual spatial aggregation, which has to be initialized as (P_1_,V_1_)by the first virtual partition P_1_ before the comparison of the noise values can be performed. As output, the set of actual partitions to be established is represented as A = [A_1_, A_2_, A_3_, …, A_m_].

Furthermore, the dynamic merging of neighboring virtual partitions was a repeated process. First, based on the temporary aggregation, the next virtual partition P_i+1_ and its associated noise value V_i+1_ was selected as targets to be merged. The value V_i+1_ was then compared with the value V_i_ of the last neighboring virtual partition P_i_ and with the maximum and minimum value of all noise values of virtual partitions in A_temp_. The next neighboring virtual partition P_i+1_ was combined into the current temporary aggregation A_temp_ if the difference between the two values was less than Min_sup. Otherwise, an actual spatial partition A_m_ was established, and a new temporary aggregation A_temp_ was initialized by (P_i+1_, V_i+1_). This process was repeated until merging of the virtual partitions on the entire segment was completed.

In this manner, the above process supported the dynamic partitioning of road segments according to the noise values at a given temporal scale. Detailed noise data sets for a given time period may be directly extracted and used as inputs for simulation-based noise mapping.

## 4. Experimental Results and Discussion

The main aim of this study was to collect and analyze participatory noise data that supported noise simulations and mapping. Using the methodology presented in the previous section, we first performed measurements and data processing to collect noise profiles, which were then fed into a simulation as the ground truth. Next, we evaluated the simulation results and noise analysis in terms of reconstruction accuracies. For example, using the campus of The Chinese University of Hong Kong as a case study, the main road segments were selected to monitor the dynamic noise data, and 20 participants joined the distributed measurements of noise data to cover the main road segments in the study area. For the noise map simulation, we used Cadna/A [5], which is a professional noise simulation software that is compatible with the GIS data, as the simulation platform.

### 4.1. Data Collection and Dynamic Processing

**Figure 4.**
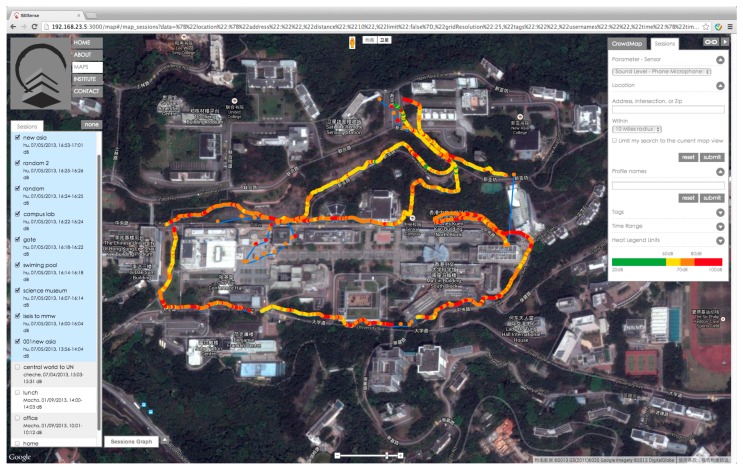
Participatory noise information stream for the main road networks collected by volunteers via the CUHK noise server.

Two-step data collection processes were performed during the experiment. We built a data server based on the open-source AirCasting [34] platform to facilitate the recording, localization and visualization of real-world noise measurements from volunteers (Figure 4). The data server was supported by a noise database, which initially stored the default noise values of the road segments to minimize biases in noise estimation caused by gaps in data collection. The default noise values were classified into four types of timescales (hours, days, months and years) and calculated as the average traffic volume for each timescale on main road segments, which were usually used to calculate traffic noise in cases of insufficient data sources.

Furthermore, a dynamic collection process of noise data was performed on the CUHK campus by 20 participants using their personal mobile phones with an app named “ISEISense”. The app is based on the AirCasting Android client, which is an open-source platform for recording, mapping, and sharing health and environmental data using smart phones. The widespread coverage in space and time has made smart phones a suitable vehicle for the collection of noise data. To facilitate the measurements in this experiment, we embedded our calibration algorithm in the ISEISense app to directly obtain accurate sample data. Once installed in the smart phones, ISEISense collects local sound level by the internal sensors (*i.e.*, microphones) on the phone. The collected data can be directly sent to the CUHK noise server via a 3G connection during the measurement session, or via WiFi at the end of the entire measurement session to reduce the energy consumption (to increase battery life). In the experiment, the time intervals for dynamic acquisition of noise measurements were 5 s, and the corresponding virtual partition was set to be 5 × 0.8 m. Each decibel measurement from the participants at the time intervals was subsequently matched to an adjacent virtual partition and stored in the noise database via the noise server.

### 4.2. Position, Estimation, and Dynamic Aggregation

**Figure 5.**
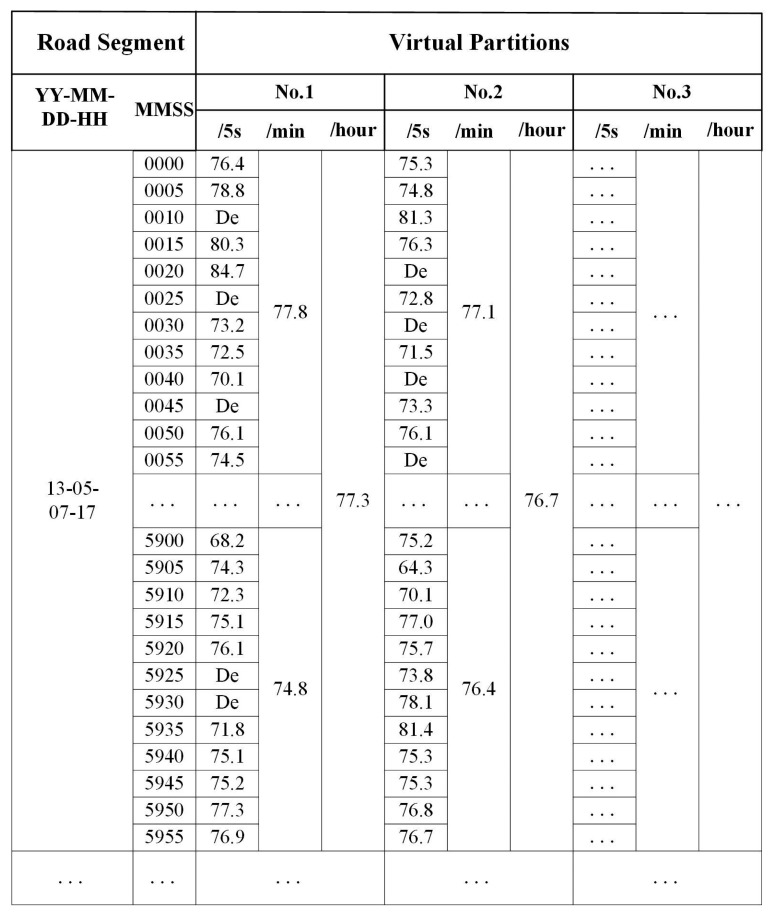
An example noise estimation for virtual partitions on road segment 15 in two periods of interest: one minute and one hour.

At a deeper level, Figure 5 shows an example of the noise estimation of each virtual partition in which all of the noise values received within a specific time period were involved. By choosing the time intervals of the dynamic acquisition of the noise measurement to be 5 s, the noise values of each virtual partition could be dynamically estimated for the time periods of interest, such as peak hours or during the daytime. Taking the virtual partitions of road segment 15 as an example, DE is the reference noise level, 74 dB, which was calculated using the traffic volume of road segment 15 from 5:00 p.m. to 6:00 p.m. Thus, DE was used as an alternative to estimate noise data when there was no feedback from the participants during this time period. To estimate the noise value for time periods of one minute and one hour, N_T_ and L_i_ were determined by taking the following time intervals in Equation (1): 12 in a 1-m period, and 720 in a 1-h period. The corresponding noise values of each virtual partition were thus dynamically estimated for time periods of one minute and one hour (see Figure 5).

**Figure 6.**
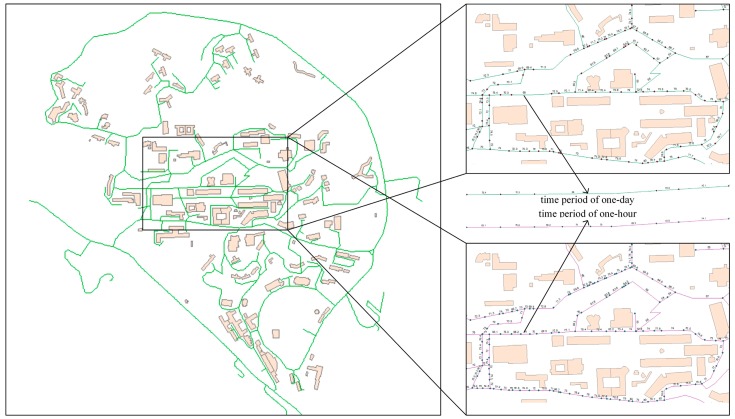
Demonstration of dynamic partitioning of the road segment on two different timescales.

Dynamic aggregation was further performed by comparing the noise values of adjacent virtual partitions and merging the noise values when the difference in their noise values was less than the threshold value (3 dB). This aggregation process generated different results according to the timescale at which the noise values were collected. For example, the dynamic partition of road segment 15 generated eight and five real partitions at timescales of one hour and one day, respectively (see Figure 6).

**Figure 7.**
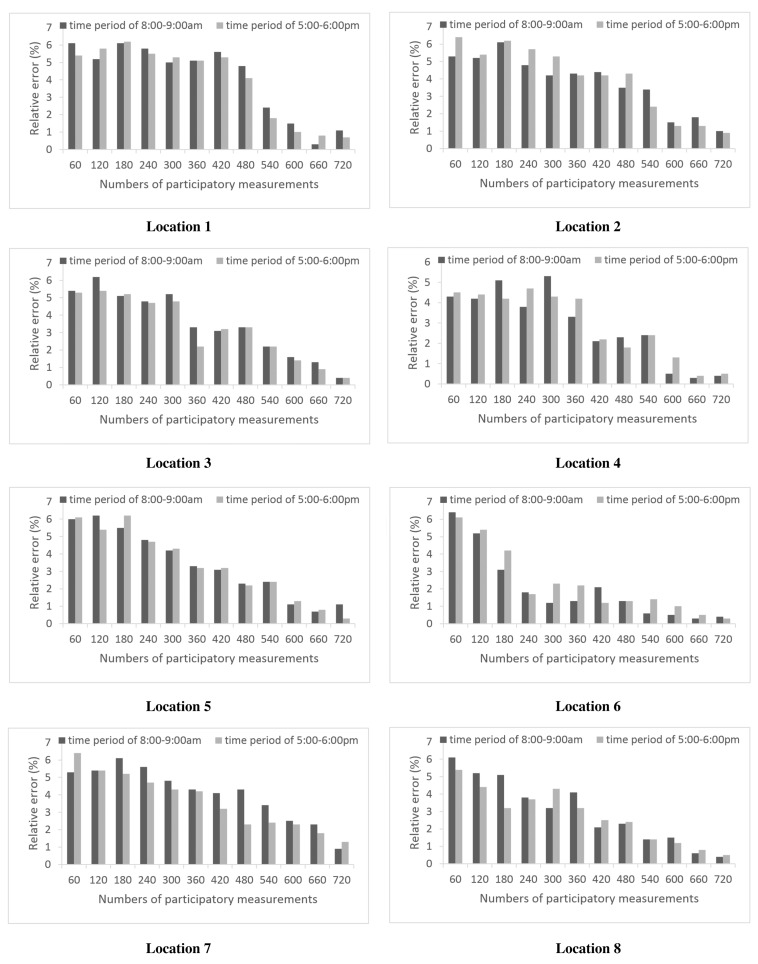
Comparison between the measured and estimated noise levels for each virtual partition at eight fixed locations.

To evaluate the accuracy of the noise estimation for different numbers of participatory measurements, we selected eight typical locations to compare the noise value between the estimation and the time-averaged field measurements, which were performed in two typical time periods of 8:00 a.m. to 9:00 a.m. and 5:00 p.m. to 6:00 p.m., for at least 30 min on 7 May 2013. The relative error between the measured and estimated values of each position at the two time periods of interest are shown in Figure 7. Analysis of the results revealed that, independent of the number of participatory measurements, the relative error was small (less than 7% (5 dB)), which demonstrated that the accuracy of the estimation was determined by the participatory noise data. With an increase in the number of participatory noise measurements, the relative error will decrease to a very small level; for example, two relative error values were approximately 0.4% for location 3 and 0.3% for location 6 during the corresponding time period. Thus, the estimated value can provide a credible overview of the actual situation when more participatory noise measurements contribute to the calculation [24].

### 4.3. Simulation-Based Noise Estimates

Based on the noise values of each dynamic aggregation of road segments during a specific time period, noise simulation and analysis was performed using the Cadna/A platform. The noise simulation examples corresponded to the noise 1.2 m above ground. From the simulation results, the following observations were made.

**Figure 8.**
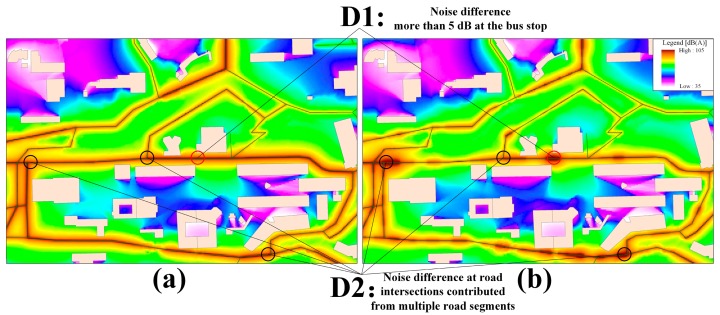
Noise simulation (**a**) based on average traffic volume and (**b**) based on the participatory noise data during a time period of one hour.

The first observation was that simulations without participatory noise data tended to neglect the local noise characteristics. This was particularly true for the noise difference found in Figure 8a,b, where Figure 8a was generated based on the average traffic volume over one hour and Figure 8b was determined by the participatory noise data during the relevant time period of one hour. For the noise difference D1, a campus shuttle bus stop appeared as an important noise source that was not considered in the simulations shown in Figure 8a. Conversely, Figure 8b showed more changes in the noise distribution near the road segment by considering this as an actual noise source, thus revealing a relative difference in noise value of greater than 5 dB. In addition, the noise difference D2 exhibited an exception at the road intersections of the main campus bus road with high traffic volume. This was because the noise of the road intersection was the collective noise contribution from multiple road segments with a number of passing vehicles, which were difficult to identify using an instantaneous measurement at one fixed location.

**Figure 9.**
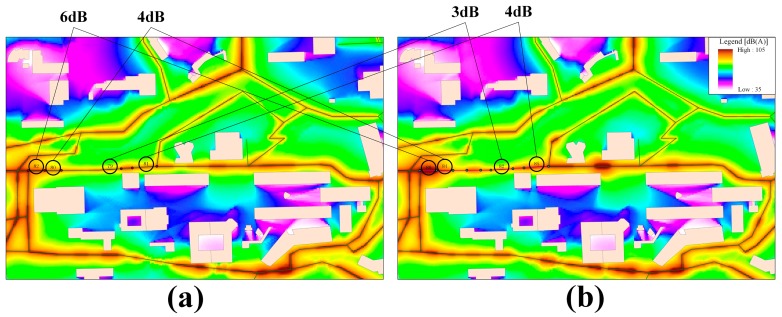
Simulation results for the noise distribution in a local area of the CUHK campus. (**a**) One day and (**b**) one hour.

Second, using participatory noise data with different spatio-temporal scales revealed extra noise disturbance on the same road segment. The simulation results based on the participatory noise values of the main road segments for two different time periods (e.g., within one day and within one hour) are illustrated in Figure 9a,b. By comparing the noise difference for the same road segment, segment 15, Figure 9a demonstrates similar noise characteristics without significant noise fluctuations during a time period of one day. In comparison, we observed that more detailed noise differences were clearly recognizable within the time period of one hour in Figure 9b. We observed that the noise values near road segment 15 exhibited multiple significant differences of 6 dB, 4 dB, 3 dB, and 4 dB. These differences were mainly due to more incidental sounds, such as traffic jams, which were recorded using participatory measurements in this road segment on the timescale of one hour. Thus, additional details regarding traffic-related noise dynamics, such as incidental sounds, which were received from participants with high granularity in space and time, have a positive contribution to the analysis of noise pollution caused by local traffic sources.

## 5. Conclusions

This study presents an integrated methodology to dynamically organize participatory noise data and to refine noise information on a linear road segment at different temporal scales. It demonstrates that a participatory and people-centric approach to noise monitoring can be used as a rational spatio-temporal data foundation to support noise simulations. In the proposed method, the dynamic organization and management of noise data are based on an analysis of participatory sensing-based noise monitoring, and it simultaneously takes road networks and spatio-temporal aggregation aspects into account. The results can be fed into the simulations as the ground truth to improve noise mapping. Some of the more important aspects of our method can be summarized as follows:

(1) Volunteered noise data collected by participants were spatially matched to the adjacent road segment, which was subdivided into multiple virtual partitions to refine the noise value at a microscopic level. Noise estimation at the spatial level of virtual partition with different temporal scales enables multiple sets of noise information to be associated with any portion of a linear road segment, further facilitating the construction of a spatio-temporal noise database.
(2) Dynamic partitioning of road segments according to noise estimation at a given temporal scale is realized via merging of adjacent virtual partitions by comparing the noise values for a specific time period. Such a dynamic aggregation process can help to identify spatial-temporal differences in noise values on the same road segment. Furthermore, the data sets maintained the consistency of noise differences, and the time periods of interest can be extracted and used as inputs for simulation-based noise mapping on the relevant timescales.
(3) Combining professional noise simulation with volunteer noise monitoring to improve geo-spatial understanding further explores a new approach to filling in the gap between participatory sensing and professional simulation, which is a sustainable strategy to enable every citizen to contribute to a collective effort to map and monitor noise pollution.

Importantly, the proposed methodology does not include all factors that have or might have an effect on participatory sensing-based noise simulations. We primarily assumed that each participant collected and sent noise data near the road and measured noise when the phone was held in a reasonable position. In fact, there are still some challenges for the current noise assessment via participatory measurements due to the participants' measurement density and the combination of different noise sources that contribute to the overall acoustical environment. In addition, although the method was used to distinguish truthful sources from volunteered observations in participatory sensing [35], filtering out faulty values, estimating accuracy, and encouraging volunteer contributors using large amounts of volunteer data are also difficult tasks. Thus, our further efforts aim to identify more detailed semantic information about the source, context or accidental nature of noise [36]. This type of semantic information will be recorded in the process of obtaining the measurements, which are vital to generate noise maps that are more useful and meaningful for end users, particularly citizens.

## Figures and Tables

**Table 1.** The algorithm to determine the set of actual partitions in a road segment.

**Algorithm 1** to determine a set of actual partitions in a road segment
**Function Determine_actual_partitions**
**Input:** V = [V_1_,V_2_,V_3_,…V_n_]; P = [P_1_, P_2_, P_3_,…P_n_]; Min_sup = minimum perceptible difference of noise value; i = 1; m = 1; A_temp_ = (P_1_,V_1_)
**Begin**
**while** (i *less than* n)
**if** (|Vi - Vi+1| *less than or equal to* Min_sup)
**if** (both |maximum noise value in A_temp_ -V_i+1_| and |minimum noise value in A_temp_ -V_i+1_| *less than or equal to* Min_sup)
push (P_i+1_,V_i+1_) into A_temp_;
i = i + 1;
**else**
push (P_i+1_,V_i+1_) into A_temp_;
m = m + 1;
i = i + 1;
**End if**
**else**
push (P_i+1_,V_i+1_) into A_temp_;
m = m + 1;
i = i + 1;
**End if**
Assign A_temp_ to A_m_;
**End while**
**End function**
**Output:** A= [A_1_,A_2_,A_3_,…A_m_]
